# MicroRNA-214 modulates neural progenitor cell differentiation by targeting *Quaking* during cerebral cortex development

**DOI:** 10.1038/s41598-017-08450-8

**Published:** 2017-08-14

**Authors:** Pengcheng Shu, Hongye Fu, Xiangyu Zhao, Chao Wu, Xiangbin Ruan, Yi Zeng, Wei Liu, Ming Wang, Lin Hou, Pan Chen, Bin Yin, Jiangang Yuan, Boqin Qiang, Xiaozhong Peng

**Affiliations:** 1 The State Key Laboratory of Medical Molecular Biology, Neuroscience Center, Medical Primates Research Center and Department of Molecular Biology and Biochemistry, Institute of Basic Medical Sciences, Chinese Academy of Medical Sciences and Peking Union Medical College, Beijing, 100005 China; 2 Department of Anatomy and Histology, Institute of Basic Medical Sciences, Chinese Academy of Medical Sciences and Peking Union Medical College, Beijing, 100005 China

## Abstract

The accurate generation of an appropriate number of different neuronal and glial subtypes is fundamental to normal brain functions and requires tightly orchestrated spatial and temporal developmental programmes to maintain the balance between the proliferation and the differentiation of neural progenitor cells. However, the molecular mechanism governing this process has not been fully elucidated. Here, we found that miR-214-3p was highly expressed in neural progenitor cells and dynamically regulated during neocortical development. Moreover, our *in vivo* and *in vitro* studies showed that miR-214 inhibited self-renewal of neural progenitor cells and promoted neurogenesis. In addition, after target screening, we identified miR-214 targets including *Quaking* (*Qki*) by binding the 3′- untranslated region (3′-UTR) of the *Qki* mRNA, which was specifically expressed in the progenitor cells of the proliferative ventricular zone as 3 *Qki* isoforms. Furthermore, overexpression and knockdown of *Qki* showed that the different isoforms of *Qki* had different functions in the regulation of neural progenitor cells differentiation. Moreover, overexpression of *Qki* could counteract the function of miR-214 in neurogenesis. Our results revealed that miR-214 maintains the balance between neural progenitor/stem cell proliferation and differentiation together with *Quaking*, its target gene.

## Introduction

Complex cerebral cortical functions, such as perception, consciousness, memory and language, depend on the precise assembly of intricate circuits by different types of neurons and glial cells. These cells are generated by neural progenitor cells (NPCs), multipotent cells of ectodermal origin that are also known as neural stem cells (NSCs), in a stereotypical temporal order with neurons first and glial cells second^1, 2^. To generate the cerebral cortex with the appropriate cellular composition and connection, the NPCs are required to maintain an exquisite balance between the proliferation and differentiation, and to modulate their competence over time^3, 4^. However, the mechanism responsible for this balance is not fully understood.

During mammalian cerebral cortical development, neural stem cells transform from neuroepithelial (NE) cells to radial glial cells (RGCs), which mainly reside in the ventricular zone (VZ) of the dorsal forebrain and generate nearly all excitatory neurons, around embryonic day (E) 9–10 in mice^2, 5^. RGCs first divide symmetrically to expand the progenitor pool and then asymmetrically to sequentially produce deep layer neurons followed by superficial layer neurons. Thereafter, the neurogenic competence of a subset of RGCs decreases, and this subset begins to produce glial cells around E16–17^1, 5^. Thus, RGCs undergo alterations in their competence accompanied by many cellular transformations to proliferate or to give rise to various types of neural cells in a temporally regulated manner. In addition, the precise spatial and temporal control of gene expression during the different phases drives these series of sequential cellular events. Extensive studies in recent years have revealed several mechanisms responsible for controlling the proliferation/differentiation choices of the NPCs, such as the bHLH family of transcription factors, which are separated into at least two major subclasses: those that promote neuronal differentiation and those that inhibit this process to maintain cells in the progenitor state^6, 7^. However, when NPCs respond to the neurogenic signals and decided to initiate the neuronal differentiation programme, expression of proneural genes begins, whereas neurogenic genes are shut down; even the existing neurogenic gene transcripts may need to be removed, which requires the exquisite control of the regulatory network. In other words, the post-transcriptional regulation plays significant roles during this process, and the molecules involved are rapidly being assessed.

MicroRNAs (miRNAs) are single stranded, endogenous, non-coding RNAs that regulate gene expression at the post-transcriptional level. MicroRNAs can form RNA-induced silencing complexes (RISCs) and can bind to target mRNAs to modulate gene expression through transcription destabilization or transcription repression^8, 9^. Many studies have focused on the regulation of gene expression by miRNAs during the process of neurogenesis^10, 11^. For example, in the developing neocortex, miR-124 was reported to inhibit NSC proliferation and promote neuronal fate by targeting PTBP1 and Sox9^12, 13^. miR-9 was proved to maintain neural progenitor proliferation and to control neuronal fate by regulating of multiple targets, including *Hes1*, *FoxG1*, and *Gsx2*, *etc*.^14–16^.

In genome-wide microarray screens for regulators of NPCs fate decision, we identified miR-214, a vertebrate-specific miRNA, that was engaged in this process to promote neurogenesis. Recent studies have shown that miR-214 operates in multiple cellular events of various organs. In zebrafish, miR-214 is expressed as early as the segmentation stage in the somite and modulates precise Hedgehog signals by targeting *su*(*fu*)^17^. MiR-214 is also involved in a feed-back loop of the Polycomb group (PcG) by down-regulating Ezh2 during the differentiation of skeletal muscle cells^18^. During the development of the retina, miR-214 has been reported to control the generation of bipolar neurons by binding to the 3′-UTRs of *Xvsx1* and *Xotx2* ^19^. Recently, miR-214 was reported to have an important role in the regulation of neuronal dendritic development^20^.

Here, we found that miR-214 is highly expressed in NPCs and dynamically regulated during neocortical development. Moreover, both our *in vivo* and *in vitro* experiments showed that miR-214 inhibits NPC cell renewal and promotes neurogenesis. In addition, after target screening, we have identified miR-214 targets such as *quaking* (*Qki*) through interactions with the 3′- untranslated region (3′-UTR) of the *Qki* mRNA, which is specifically expressed in the NPCs of the proliferative VZ; all 3 *Qki* isoforms are expressed. Our study shows that with its functionally relevant target gene *Qki*, miR-214 plays a crucial role in NPC fate determination during cerebral cortex development.

## Results

### miR-214 is Abundantly Expressed in NPCs and Neurons during Neurogenesis

To investigate the miRNA profile in the process of mouse cerebral cortex development, we extracted total RNA from the dorsal regions of fetal mouse cerebral cortexes at different stages (E12.5, E14.5, E16.5 and E18.5), and analysed the miRNA expression level using the miRNA array based on the miRBase Database Release 19.0 (http://www.mirbase.org/) (data not shown). We selected miRNAs with signal intensities on E12.5 that were higher than 1000 and showed a descending expression tendency during the process of cerebral cortex neurogenesis. miR-214, also called miR-214-3p, was among the 26 microRNAs we selected.

**Figure 1:**
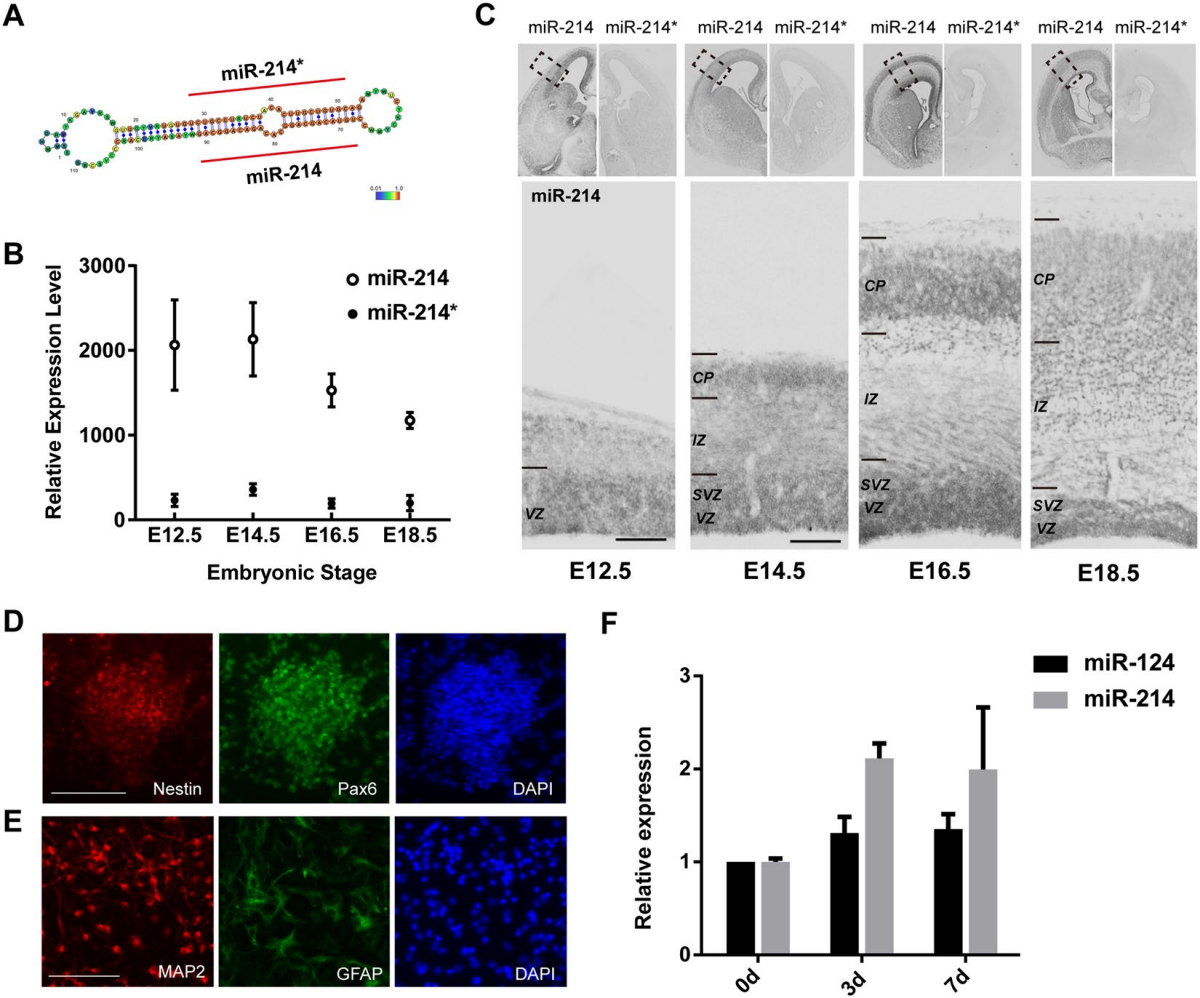
Expression pattern of miR-214 in the developing neocortex and cultured neural stem cells. (**A**) Predicted secondary structure and sequence conservation of the miR-214 precursor hairpin. The maturation regions of miR-214 and miR-214* are marked with red straight lines, and the colour of the base represents its conservation with higher conservation in red and lower in blue (modified from Rfam database 11.0). (**B**) Microarray analysis of the expression level of the mature miR-214 and miR-214* in the mouse embryonic cerebral cortex. Total RNAs of the E12.5, E14.5, E16.5 and E18.5 dorsal cortexes were extracted and tested by the LC Sciences company. Error bars show the standard error of mean. (**C**) The expression dynamics of miR-214 and miR-214* in the mouse embryonic cerebral cortex. *In situ* hybridization for miR-214 and miR-214* on coronal sections of the embryonic telencephalon. The top left panel of each stage shows the expression of miR-214, and the top right panel shows that of miR-214*. The bottom panel shows the corresponding neocortical areas of the black dashed boxes of the top left panel at a higher magnification. VZ, ventricular zone; SVZ, sub-ventricular zone; IZ, intermediate zone; CP, cortical plate; the scale bar in **C** is 100 μm. (**D**,**E**) Immunofluorescence of NSCs during proliferation conditions (**D**) and the differentiation stage (**E**). (**D**) Staining of the neural sphere with neural stem cell markers Pax6 (green) and Nestin (red) (**E**) Immunofluorescence of differentiated cells 3 days after induction with neuron marker MAP2 (Red) and astrocyte marker GFAP (green). Scale bar: 100 μm. (F) Expression levels of miR-214 and miR-124 before and after differentiation of NSCs. Total RNA of the NSCs and differentiated cells was extracted and tested by real-time PCR, and the miR-214 signal was normalized to that of the U6 snRNA (n = 3, p = 0.035).

MiR-214 is a mammalian conserved miRNA generated by the mir-214 gene, which is positioned within the introns of the *dynamin 3* (*Dnm3*) gene in mice. Like most other miRNA genes, the mir-214 gene produces a transcript known as pre-mir-214 that can be processed into two different mature miRNAs—miR-214 and miR-214*, which is also known as miR-214-5p. Microarray analysis revealed that miR-214 was expressed in a moderate and descending level during the neurogenesis of the cortex, but miR-214* expression was very low (Fig. 1A,B).

To gain further insight into the spatial and temporal expression pattern of miR-214, we performed *in situ* hybridization studies with locked nucleic acid (LNA) -modified probes^21^ that were specific for miR-214 and miR-214* at four stages of developing brain. We used miR-124, a neuron-enriched miRNA, as a control. The results showed that miR-124 was expressed in both the migrating neurons in the subventricular zone (SVZ) and intermediate zone (IZ) and the mature neurons in the cortical plate (CP), but not in the progenitor cells of the VZ (Supplemental Fig. 1A,B). miR-214 also maintained a relatively high expression level between E12.5 and E18.5, the stage at which the NPCs were proliferating and sequentially differentiating into neurons and glial cells, and readily detected in the CP where the neurons were located. However, miR-214 that was expressed in the VZ where the NPCs resided during the developmental stages were analysed, unlike miR-124 (Fig. 1C). For most of the neurons in the CP are originally derived from the NPCs of the VZ, thus, these results indicate that miR-214 is expressed in the NPCs of the VZ, and maintains its expression in the post-mitotic neurons.

By contrast, miR-214* showed a low signal near or below the detection limit (Fig. 1C) under the same ISH condition, and signal development with miR-214. Although the probability that the miRNA precursor duplex could give rise to two different mature miRNAs is equal in theory, it is possible that only one strand survives or has functions^22, 23^. This indicates that miR-214 is the functional guide strand of the miR-214 duplex, which is complementary to the target, while miR-214* is the passenger strand, which is subsequently degraded.

### Increasing the Expression of miR-214 during NPCs Differentiation *in vitro*

As miR-214 is expressed in both NPCs and cortical neurons, to further verify the miR-214 expression dynamic during NPCs differentiation, we used an *in vitro* primary NPCs culture system to analyse the miR-214 expression profile. The primary embryonic NPCs were freshly derived from E14.5 mouse dorsal forebrains, and most of the cultured cells formed neurospheres and expressed neural stem cell marker Pax6 and Nestin under maintenance conditions (Fig. 1D). When induced in differentiation conditions, the cells immediately expressed the neuron marker, microtubule associated protein 2 (MAP2), or glial fibrillary acidic protein (GFAP), an astrocyte marker, three days after induction (Fig. 1E). Then, we extracted the total RNA from the NPCs and differentiated neural cells respectively, and measured the expression dynamics of miR-214 by real-time PCR. As shown in the Fig. 1F, the expression level of miR-214 increased along with the differentiation of the NSCs (Fig. 1F), as did miR-124, suggesting that miR-214 play a role in neuronal differentiation.

### MiR-214 Promotes Neurogenesis both *in vivo* and *in vitro*

**Figure 2:**
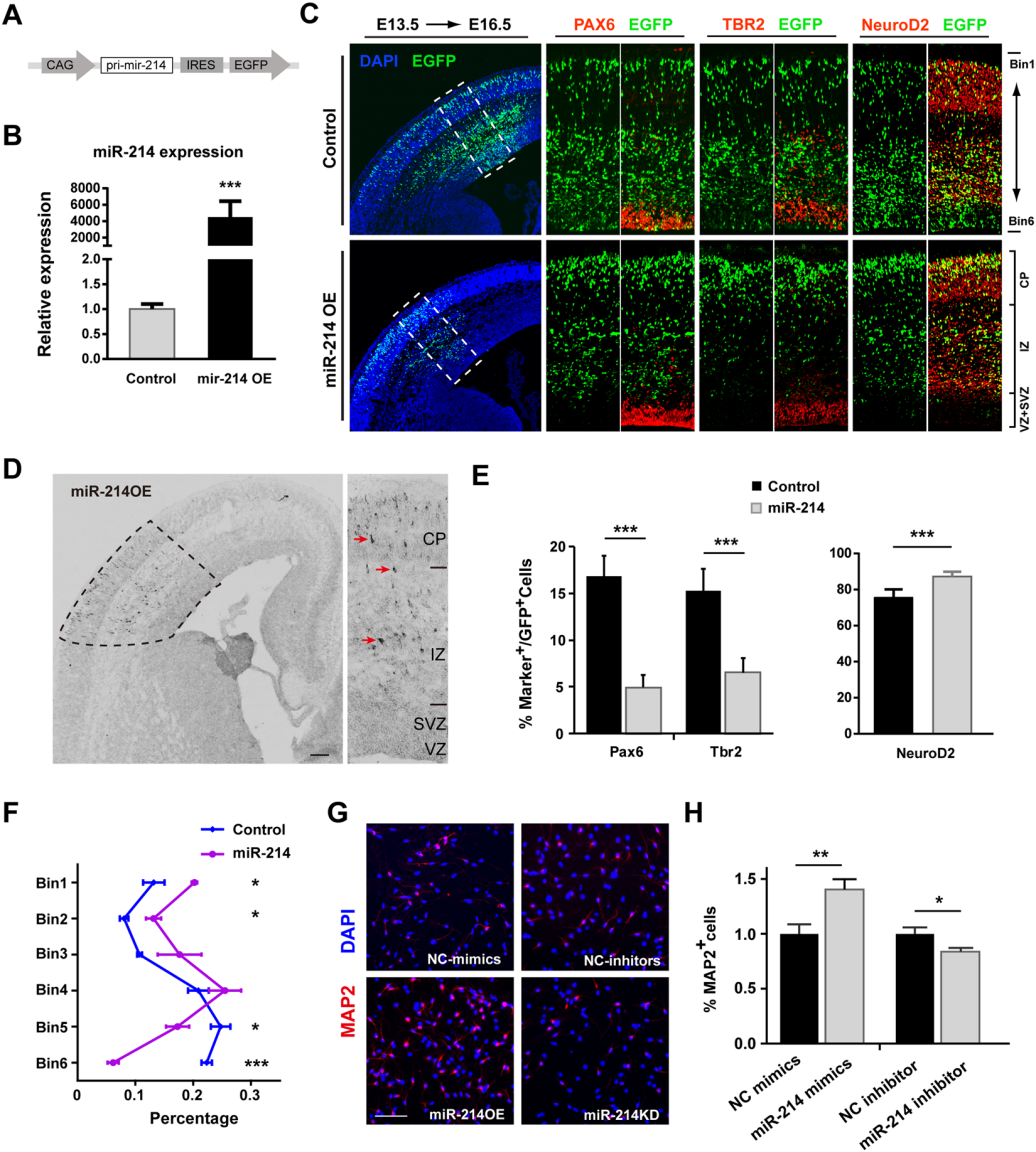
miR-214 promotes the differentiation of neural progenitor cells and NSCs. (**A**) The miR-214 overexpression construct. The miR-214 precursor hairpin sequences and both side flanking sequences were cloned and inserted into the pCIG vector. (**B**) Quantitative real-time PCR analysis of the overexpression of miR-214 in HEK-293ET cells 48 hours post-transfection. The expression of miR-214 was normalized to that of the U6 snRNA (n = 4 independent biological repeats). Error bars show the standard error of mean, and the comparisons were performed by Student’s t-test; the statistically significant P values are shown as ***(<0.001). (**C**–**F**) Sections of the E16.5 forebrains electroporated at E13.5 with plasmids expressing GFP alone (top panel) and those co-expressing miR-214 (bottom panel), and double-labelling was used to detect the expression of Pax6 (**C**, left panel), Tbr2 (**C**, middle panel) or NeuroD2 (**C**, right panel) in GFP^+^ cells. *In situ* hybridization was used to examine the expression of the exogenous miR-214 in **D**. The dashed line in the left panel indicates the region where the transfected cells are distributed, and the arrows in the right panel showed the overexpression signal in a detailed view. CP, cortical plate; IZ, intermediate zone; SVZ, subventricular zone; VZ, ventricular zone. (**E**,**F**) Quantitative analysis of the E16.5 dorsal forebrains for the fraction of GFP^+^ cells co-expressing the markers (**E**) and for the distribution of those by dividing the neocortex into 6 equal bins (**F**) within the transfected neocortex (n = 5 brains from 3 surgeries for the control and 4 brains from 3 surgeries for miR-214 OE). Error bars show the standard deviation, and the comparisons were performed by Student’s t-test. (**G**,**H**) miR-214 stimulates the neuronal differentiation of cultured NPCs. Cells were transfected with miR-124 mimics, the miR-124 inhibitor or the related control and induced with RA. Two days after treatment, the cells were fixed and stained with neuron marker MAP2. Scale bar: 100 μm. (**H**) The ratio of the MAP2 positive cells in the miR-214 mimics, miR-214 inhibitor and control groups (n = 10, **p = 0.005). Error bars show the standard error of mean, and the comparisons were performed by Student’s t-test, the statistically significant P values are shown as *(<0.05), **(<0.01) or ***(<0.001).

To investigate the possible roles of miR-214 in NPCs fate determination in the cerebral cortex, we cloned pri-miR-214 with its flanking sequence and inserted it into the pCIG vector, which was driven by a chicken β-actin promoter and followed by an IRES initiated EGFP coding sequence (Fig. 2A); hence the cells transfected with the miR-214 overexpression plasmid could be tracked by GFP fluorescence. We first confirmed the overexpression of miR-214 by real-time PCR after transfection of this plasmid into HEK-293ET cells. As shown in the Fig. 2B, the relative expression of mature miR-214 in cells transfected with the miR-214 overexpression plasmid was 4000-fold higher than the control group (Fig. 2B).

Then, we used *in utero* electroporation (IUE) to introduce plasmids expressing GFP alone (control) or with overexpression of exogenous miR-214 (miR-214OE) into the dorsal forebrain at E13.5, and collected the embryos 72 hours later at E16.5. Transfection of miR-214 led to a significant decrease in the proportion of GFP^+^ cells in the VZ and an increase in the cells that migrated to the CP compared with the control (Fig. 2C). Moreover, we used *in situ* hybridization to assess the expression of the exogenous miR-214 directly. In the E16.5 neocortex, the cells with strong signals were mainly distributed in the cortical plate, consistent with the distribution patterns of the GFP^+^ cells (Fig. 2D). To quantify this, we subdivided the neocortices into 6 equally sized subintervals (bins), with bin 1 being the most superficial layer I and bin 6 being the ventricular surface. There were significantly more GFP^+^ cells in the bins 1 and 2 with fewer in the bins 5 and 6 (Fig. 2F). We further examined the fate of GFP^+^ cells by co-staining with RGC marker Pax6, INP marker Tbr2, and projection neurons marker NeuroD2, and found a significant decrease in the proportion of Pax6^+^ GFP^+^ and Tbr2^+^ GFP^+^ cells, but an increase in the proportion of NeuroD2^+^ GFP^+^ cells, among the total GFP^+^ cells, compared with the control (Fig. 2E). Thus, these results suggest that miR-214 promotes the NPCs differentiation to neurons.

To further validate the function of miR-214 in neural progenitor cells, we transfected NPCs *in vitro* with miR-214 mimic or inhibitor to overexpress or knockdown miR-214 respectively. To avoid the cell death caused by complete withdraw of Epidermal growth factor (EGF) and Fibroblast growth factor (FGF), and the irreversible differentiation promoted by Foetal Bovine Serum (FBS), we used 2 ng/ml cytokines and 1 μM retinoic acid (RA) to initiate cell differentiation at the same time. Forty-eight hours after miR-214 mimic transfection, the ratio of differentiated neurons increased compared with the control group, while using the miR-214 inhibitor for functional knockdown(KD) of miR-214 had the opposite effect (Fig. 2G,H). These results are consistent with the *in vivo* phenotype and indicate that miR-214 reduces NPC proliferation and enhances neuronal differentiation.

### QKI is a Direct Target of miR-214 during Neural Progenitor Cell Differentiation

**Figure 3:**
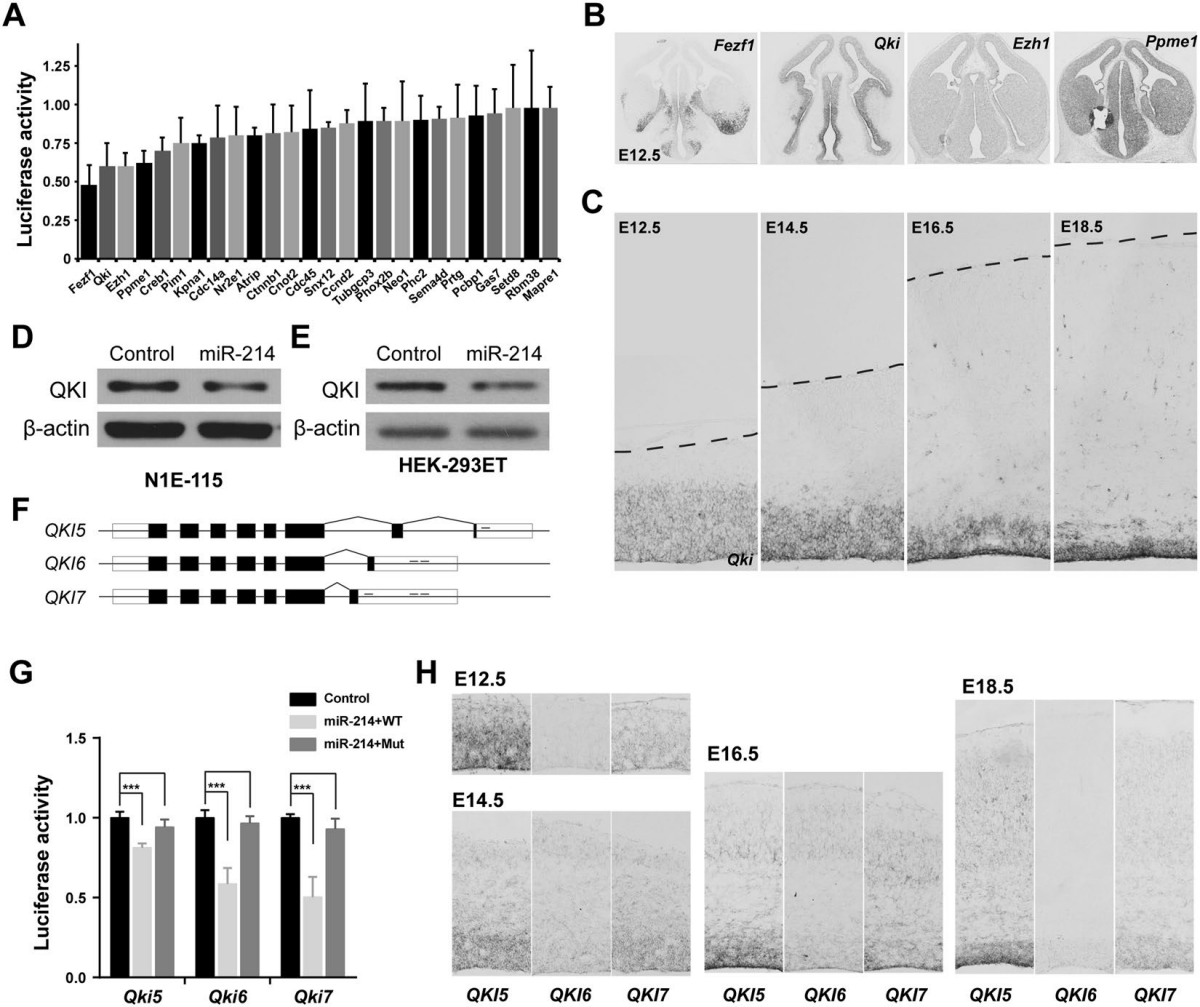
*Qki* is expressed in neural progenitor cells and targeted by miR-214. (**A**) Screening with 3′UTR luciferase reporter assays to determine functional miR-214 binding sites. Bar graphs show the relative luciferase activity of 26 potential target gene 3′-UTRs in HEK-293ET cells after overexpression of miR-214. Data were normalized to Renilla luciferase activity. (**B**) The mRNA expression of *Fezf1*, *Ezh1*, *Qki* and *Ppme1* in the mouse telencephalon at E12.5, performed by *in situ* hybridization. (**C**) The dynamics of *Qki* mRNA expression during cerebral cortex development at E12.5, E14.5, E16.5 and E18.5. (**D**,**E**) Western blot analysis of QKI expression after overexpression of miR-214 for 48 hours in N1E-115 cells (**D**) and HEK-293ET cells (**E**). β-actin was included as a loading control. Uncropped versions of all western blots are shown in Supplementary Figure 3. (**F**) The structures of the three major alternative splice variants of *Qki* and the predicted miR-214 binding sites in their 3′ UTRs. The “−” sign over the black line represents the possible miR-214 binding sites. (**G**) Dual luciferase assays of 293ET cells co-transfected firefly luciferase construct containing the wild-type(WT) or respective target sites mutant(Mut) 3′-UTR of the *Qki* isforms, along with the miR-214 expression plasmid or the empty vector. All experiments were normalized to co-transfected Renilla luciferase. Histograms show normalized mean values of the relative luciferase activity, from three or more independent transfections. Error bars show the standard deviation, and the comparisons were performed by Student’s t-test; the statistically significant P values are shown as *(<0.05), **(<0.01) or ***(<0.001). (**H**) The *in situ* hybridization of the three *Qki* variants with specific probes respectively shows their expression pattern in the cerebral cortex at E12.5 to E18.5. Scale bar: 100 μm.

miRNAs are generally considered to alter target gene expression levels by affecting the stability or the translation of “targeted” mRNAs. Therefore, to further elucidate the mechanisms responsible for the promotion of neurogenesis by miR-214, we attempted to identify the regulatory partners of miR-214 during NPCs differentiation. First, we used prediction algorithms including TargetScan and PicTar to search for target genes, the 3′-UTRs of which might interact with miR-214. We then selected the genes that had been previously reported to be related to cell fate determination or neural development. From these analyses, we selected 26 candidate genes for further study (Fig. 3A). Then, we performed dual-luciferase reporter assay to validate whether these genes were bona fide and direct targets of miR-214. To this end, we generated luciferase reporters with these 26 mouse genes 3′-UTRs including their complementary sequences, some of which were full-length of their 3′-UTRs. The luciferase assays demonstrated that some genes were down-regulated by miR-214, as shown in Fig. 3A. We selected four genes (Fezf1, Ezh1, Quaking and Ppme1) that reduced the luciferase activity to below 70% for further analysis.

Next, we performed *in situ* hybridization experiments to analyse the spatiotemporal expression of these four target genes between E12.5 and E18.5, and found that *Quaking* (*Qki*) was mainly expressed in the neural progenitor cells of the cerebral cortex (Fig. 3C), while the other three genes were mainly expressed outside the cerebral cortex (such as *Fezf1* at E12.5) or broadly expressed in the cerebral cortex (Fig. 3B and Supplemental Fig. 1C,D). These results indicate that *Qki* may play roles in NPCs during neurogenesis. Thus, we concentrated on *Qki* to further verify whether it was a functional target of miR-214 in NPCs during neurogenesis.

To determine whether miR-214 could regulate endogenous *Qki*, we assessed the QKI protein expression levels after miR-214 overexpression in mouse N1E-115 and P19 cells and in human HEK-293ET cells. In these three cell lines, transfection with synthetic miR-214 mimics led to reductions in the endogenous QKI protein, indicating that miR-214 could repress the expression of the QKI protein *in vitro* (Fig. 3D,E and Supplemental Fig. 2A).

### Different Isoforms of *Qki* have Different Functions in Neurogenesis

QKI is an RNA-binding protein that belongs to the signal transduction and activation of RNA (STAR) family and is encoded by the *qk* gene, the coding sequence and genomic organization of which are highly conserved in mammals. At least three major alternatively spliced isoforms of *Qki* are generated, and they encode QKI5, QKI6 and QKI7, named based on the lengths of the *Qki* mRNAs and their C-terminal 30 amino-acids differences. Interestingly, three splice isoforms of the mouse Qki have two entirely distinctive but conserved 3′UTRs: one is used by *Qki5* and one is shared by *Qki6* and *Qki7*. All three major splice isoforms contain one or more miR-214 binding sites (Fig. 3F). Although the repressive activity of miR-214 differs among them, the luciferase activities of three isoforms of *Qki* 3′UTR are marked decrease of in the expression of miR-214, and mutating these binding sites abolished the repression by mir-214 (Fig. 3G a﻿nd Supplemental Fig. 2B). Using *in situ* hybridization, we confirmed that all three *Qki* splice isoforms were mainly expressed in the VZ, with a higher intensity of *Qki*5 than the other two isoforms, *Qki*6 and *Qki*7 (Fig. 3H).

**Figure 4:**
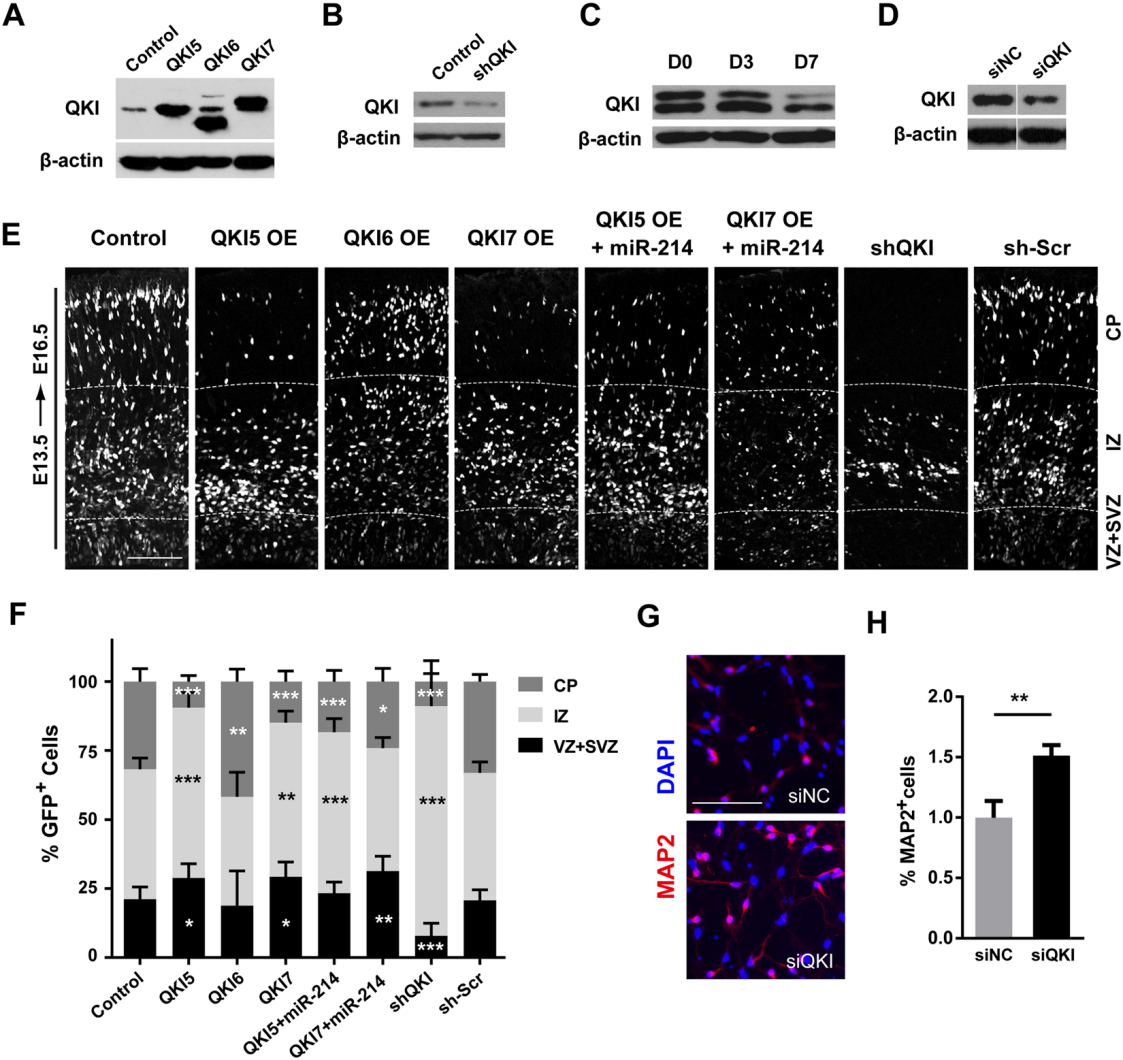
QKI suppresses differentiation of NPCs both *in vitro* and *in vivo*. (**A**,**B**) Western blot analysis of the overexpression of the three *Qki* isoforms (A) and the knockdown of the QK protein with the sh*Qki* plasmid (**B**) after transfection for 48 hours. (**C**) The QKI protein expression dynamics during the differentiation of the NSCs. (**D**) For the knockdown of QKI with *Qki* siRNA, the samples were derived from the same experiment, and the gels/blots were processed in parallel. All analyses were normalized to β-actin as a loading control. Uncropped versions of all western blots are shown in Supplementary Figure 3. (**E**) Sections of the E16.5 forebrains electroporated at E13.5 with plasmids expressing GFP alone or those co-expressing each isoform of Qki, the sh-Scramble (sh-Scr) and sh-*Qki* plasmid, the QKI5 plus miR-214, or QKI7 plus miR-214. CP, cortical plate; IZ, intermediate zone; SVZ, subventricular zone; VZ, ventricular zone. (**F**) Quantitative analysis of the E16.5 dorsal forebrains for the distribution of the GFP^+^ cells in E. The GPF^+^ cells in the VZ plus SVZ, IZ and CP of the cerebral cortex were counted to perform a statistical analysis. Error bars show the standard deviation, and the comparisons were performed by Student’s t-test. the statistically significant P values are shown as *(<0.05), **(<0.01) or ***(<0.001). (**G**,**H**) Cultured neural stem cells were transfected with the *Qki* siRNA or scramble control. Two days after transfection, the cells were fixed and stained with neuron marker MAP2. Scale bar: 100 μm. (**H**) The ratio of MAP2 positive cells in the *Qki* siRNA (siQKI) and scramble control (siNC) groups (n = 5, p = 0.017). Error bars show the standard error of mean, and the comparisons were performed using Student’s t-test; the statistically significant P values are shown as *(<0.05), **(<0.01) or ***(<0.001).

Then, we cloned the coding sequence of three transcript variants into the pCIG vector respectively and confirmed protein overexpression by western blot (Fig. 4A and Supplemental Fig. 2C). Then, we performed *in utero* electroporation to investigate whether the different alternative isoforms tended to mediate similar or distinct functions in regulating NPCs proliferation/differentiation balance. By introducing each isoform of *Qki* into the dorsal forebrain at E13.5, we found that overexpression of QKI5 and QKI7 caused significant decreases in the proportion of GFP^+^ cells in the CP, which was accompanied by increases in the cells that resides in the VZ and SVZ, compared with the control (Fig. 4E). When the neocortices were subdivided into 6 equally sized subintervals (bins), as described above, the GFP^+^ cells were significantly more localized to bins 5–6 and less so to bins 1–2 (Fig. 4F). Interestingly, overexpression of QKI6 showed no significant change in this process, with a slight increase in bins 1–2. We further evaluated the fate of the GFP^+^ cells by immunostaining for Pax6, and found that overexpression of QKI5 and QKI7 increased the proportion of Pax6^+^ cells among all transfected cells significantly, compared with the control (Supplemental Fig. 2D). Thus, these results indicate that the different isoforms of *Qki* have different functions in neurogenesis: QKI5 and QKI7 repress neural differentiation, while QKI6 can not.

### The Function of Qki is Opposite to That of miR-214 in NPCs Fate Determination

To further analyse whether QKI is necessary for neurogenesis, we performed a loss-of-function assay using the Qki-shRNA construct as described previously^24^ (Fig. 4B). First, we validated the knockdown efficiency of shQki by immunoblotting and observed an obvious reduction in QKI expression after introducing the shRNA (Fig. 4B). Then, we electroporated the shQki or sh-Scramble (sh-Scr) at E13.5, and harvested the embryos at E16.5. In the control neocortex transfected of sh-Scr plasmids, the GFP^+^ cells were broadly distributed amongst the VZ and the CP, while knockdown of QKI strongly decreased the proportion of GFP^+^ cells in the VZ and the CP, which was accompanied by greater accumulation in the IZ (Fig. 4E,F). In other words, knockdown of QKI promoted the exit of cells from the neural stem cell layer, in a similar manner to miR-214 overexpression, but showed a stronger phenotype than the latter. As showed in Figure 3D,3E and Figure 4B, using the same amount of the shQKI, it showed stronger inhibitory effects than miR-214OE (shQKI 42.5 ± 5.1% vs. 66.3 ± 0.8% miR-214), by quantification of fold change of QKI expression though ImageJ. Thus, it shows the effect of shQKI or miR-214 on neurogenesis would be determined by the ratio of the decreasing of endogenous QKI, and this linear correlation between expression and the phenotype further confirms the role of QKI in neurogenesis.

Furthermore, we also analysed the expression of the QKI protein in NSCs during the process of differentiation, and the results showed that QKI expression decreased as a consequence of differentiation (Fig. 4C). Then, we used QKI siRNA to knock down QKI *in vitro* (Fig. 4D) and found that knockdown of QKI in NSCs could increase neuron differentiation in a consistent manner to the function of miR-214 (Fig. 4G,H). Therefore, ectopic QKI5 and QKI7 inhibited neurogenesis and neuronal migration, whereas knockdown did the opposite.

### Qki Acts Downstream of miR-214 during Neurogenesis

To test whether QKI was a functional target for miR-214 to trigger neurogenesis, we performed an *in vivo* rescue assay to test of the functional equivalence of miR-214 and QKI. When electroporation was performed with equal amounts of QKI5 or QKI7 with the miR-214 overexpression plasmid at E13.5, the GFP^+^ cells were broadly distributed amongst the VZ and the CP, in a similar manner to the control group. This indicates that *QKI* overexpression can partially rescue the effect of miR-214 in neural progenitor cell differentiation (Fig. 4E,F).

## Discussion

Gene expression in neural progenitor cells is strictly regulated in a spatial- and temporal-specific manner, and as an important post-transcriptional regulator, miRNAs are heavily involved in cerebral cortex development. In this study, we have demonstrated an essential role for miR-214 in the neural progenitor cell proliferation and differentiation decision.

First, miR-214 is expressed in the VZ, where the NPCs reside, and the CP, where the differentiated neurons are located, in the cerebral cortex from E12.5 to E18.5. Therefore, miR-214 might be expressed in the NPCs lineage both before and after differentiation. In a further analysis of the *in vitro* cultured NPCs, the abundance of miR-214 increased as the NPCs differentiated into the neurons. These results indicate that miR-214 might play a role in the process of neural differentiation. Second, the *in vivo* experiments show that miR-214 overexpression leads to the exit of the electroporated cells from the stem cell zone and their differentiation into neurons. Additionally, in cultured primary NPCs, ectopic miR-214 reduces the self-renewal capacity of the neurospheres and promotes neuronal differentiation. These results show that miR-214 functionally increases neurogenesis. Lastly, miRNAs often coordinate different cell activities via interactions with numerous target genes. Based on the gene expression and interaction activity with miR-214, we identified *Quaking* (*QKI*) as a direct target of miR-214. Further analysis showed that Qki is involved in the process of neurogenesis, as discussed below. Notably, miR-214 is also reported to regulate multiple genes, such as *Ezh2* and *su*(*fu*), which are widely recognized as molecules that play roles in cell fate decisions. Thus, these data collectively support the evidence that miR-214 plays a role in neurogenesis during cerebral cortex development.

*QKIs* are RNA-binding proteins encoded by the *qk* gene, and they belong to the signal transduction and activation of RNA (STAR) family, which are the key regulators that link external signals directly to RNA; they are involved in RNA stability and splicing and thus effectively help to maintain the dynamic and progressive equilibrium of the development process^25, 26^. In vertebrates, *Qk* is essential for early development, and some mutations in *qk* can cause embryonic lethality^27, 28^. The functions of *Qk* have been revealed first by a spontaneous mutant mouse, known as the *Quaking mouse* (*Qk* ^*v*^), with deficient myelination in the central nervous system^29^. In the past few years, extensive studies have also shown that the QKI proteins are expressed in differentiated glia and have been implicated as regulators to control the proliferation and differentiation of myelinating glial cells^24, 30, 31^. However, the QKI proteins are initially detected in the neural progenitor cells of the VZ during neurogenesis^32^. Moreover, expression is selectively silenced in the neuronal lineage and maintained in glia during neuron-glial cell fate decision^32, 33^. Thus, the QKI proteins are postulated to participate in neural cell fate specification, but there is no experimental evidence to support this. In this study, we directly demonstrated the functional requirement of QKI in this process. Overexpression of two isoforms of QKI, QKI5 and QKI7, increased the proportion of cells in the VZ at the expense of the cells in the CP. Using reciprocal tests to assess the necessity of QKI for maintaining NPCs fate, evidences from *in vitro* cultured neurospheres and *in vivo* electroporation show that knockdown of QKI decreased the number of NPCs in the VZ and the co-expression of RGC marker Pax6. Furthermore, *Qki* is a direct target and functional opposite of miR-214. These results support the notion that QKI contributes to NPC cell fate decisions to help maintain NPCs stemness. Notably, QKI may also influence neuronal migration, as knocking down QKI decreases the proportion of cells reaching the CP.

The *qk* gene can produce three predominant *Qki* isoforms by alternative splicing of the 3′-coding exons. Different isoforms code three different proteins, namely, QKI5, QKI6 and QKI7 respectively, which are termed based on the length of the *Qki* mRNAs. All QKI proteins share the same STAR domain in the N-terminus, which is responsible for RNA-binding^34^, and differ only in their C-terminus and 3′-UTR sequence. The distinct C-terminus of the QKI isoforms determine their subcellular localizations: QKI5 is predominantly nuclear localization for its C-terminus harbours a nuclear localization signal (NLS), and QKI6 and QKI7 are cytoplasmic^35^. The different subcellular localizations of the QKI isoforms indicated their differential functions. For example, *Qki6* and *Qki7* are gradually increased during myelination, while *Qki5* declines. However, QKI5 is likely to be responsible for the early embryonic development. Interestingly, we also found that these 3 splice variants of *Qki* showed different expression patterns in the developing cerebral cortex, while *Qki5* and *Qki7* are specifically expressed in the ventricular zone of the cerebral cortex during neurogenesis. Using functional studies, we also found that QKI5 and QKI7 are the functional isoforms of QKI in NPC fate determination. Moreover, these two entirely different long 3′-UTRs are highly conserved among vertebrate species. Taking account of the diverse inhibitory activities of miR-214, we hypothesized that miR-214 may be involved in the regulation of the dynamic among the QKI splice variants.

Taken together, our data suggest that miR-214 and its target gene, *Qki*, are crucial regulators of cell differentiation in the developing cerebral cortex, during which miR-214 represses *Qki* expression to modulate neural progenitor cells differentiation. Notably, miR-214-mediated negative regulation of *Qki* has recently been reported in other cellular process, such as angiogenesis^36^, neuronal dendritic morphogenesis^20^, and smooth muscle cell differentiation^37^. Interestingly, accumulating data support the notion that QKI is involved in multiple neurological disease, such as schizophrenia^38, 39^, ataxia^40^, and other diseases. This indicates that a more comprehensive regulatory relationship between miR-214 and *QK* is involved in several applications.

## Methods

### Animals

All animals used for *in vivo* experiment were 8–12 weeks-old CD1 mice from the Beijing Vital River Laboratory Animal Limited Company. Mice were maintained in the Animal Centre of Peking Union Medical College. Animal care and experiment were approved by the Institutional Animal Care and Use Committee of the Chinese Academy of Medical Sciences and Peking Union Medical College with all procedures in compliance with the Experimental Animal Regulations (China Science and Technology Commission Order No. 2).

### Cell culture and transfection

The HEK-293ET cell line was provided by Dr. Chengyu Jiang (Peking Union Medical College) and cultured in complete Dulbecco’s Modified Eagle’s Medium (DMEM) (Thermo Scientific) with 10% (v/v) foetal bovine serum (FBS). The N1E-115 murine cell line was provided by Dr. Yan Zhou (Wuhan University) and maintained in Dulbecco’s modified Eagle’s medium (DMEM) with 4.5 g/L glucose, without sodium pyruvate, and with 10% foetal bovine serum (HyClone Laboratories).

Neural progenitor cells were isolated from the E14.5 mouse forebrain and digested into single cell suspension with Accutase (Sigma), and cells were cultured in DMEM/F12 proliferation medium supplemented 2% B27 supplement, 20 ng/ml EGF, 20 ng/ml bFGF and 0.2% BSA for proliferation. For differentiation assays, NPCs were dissociated into single cells and cultured in DMEM/F12 supplemented with B-27 and 10% FBS or 1 μM retinoic acid (RA). Cells were allowed to differentiate for 3 or 7 days, followed by further analysis. All cell lines were cultured at 37 °C with 5% CO_2_.

For transfections, plasmids were transfected into HEK-293ET and N1E-115 cells with a final concentration of 1.6 μg/ml using Lipofectamine 2000 (Invitrogen). siRNA and miRNA mimics or inhibitors, which are 2′-O-methoxyethyl (2′-MOE) modified anti-miRNA oligonucleotides (AMOs), were purchased from the Shanghai GenePharma Company and transfected into primary NPCs with final concentrations of 50 nM using INTERFERin (Polyplus) according to the manufacturer’s protocol.

### *In situ* hybridization (ISH)

Timed pregnant mouse embryonic brains were fixed in 4% paraformaldehyde (PFA) in phosphate buffered saline (PBS). The fixed tissues were cryoprotected with 25% sucrose in PBS and equilibrated in the O.C.T. Compound (Sakura) for 15–30 min before freezing. Sixteen-micron cryosections were generated and stored at −20 °C. Probes of miR-214, miR-214* and miR-124 labelled with digoxigenin (DIG) for *in situ* hybridization were purchased from Exqion. For the synthesis of probes of the protein coding genes, the unique sequences of the genes were cloned into the pGEM-T vector. The digoxigenin (DIG)-labelled antisense and sense cRNA probes were synthesized using T7 or SP6 RNA polymerase (New England Biolabs) or T3 RNA polymerase (Roche Diagnostics) *in vitro* with the corresponding plasmids using the DIG RNA Labeling Kit (Roche Diagnostics) according to the manufacturer’s instructions. The *QK* probes were designed from the unique coding sequences of the three mRNA isoforms respectively. The ISH procedure was performed as described^21^.

### Real-time PCR analysis

Total RNA was extracted from cells using the Trizol reagent (Invitrogen) and the concentration was calculated with the NanoDrop ND-2000. For mRNA semi-quantitative PCR, 2 μg of total RNA was reverse-transcribed to cDNA with reverse transcriptase (New England Biolabs) following the manufacturer’s protocols. Real-time PCR was carried out using the SYBR-green-containing PCR kit (Takara) according to the manufacturer’s instructions. MiRNA real-time PCR was carried out as previously described^41^. The stem loop RT PCR primer sequences were as follows: miR-214-RT-(5′-GTCGTATCCAGTGCAGGGTCCGAGGTATTCGCACTGGATACGACACTGCC-3′) miR-214-realtime-Forward-(5′-CGCCGACAGCAGGCACA-3′), U6-RT-(5′-GTCGTATCCAGTGCAGGGTCCGAGGTATTCGCACTGGATACGACAAAAATATG -3′), U6-realtime-Forward-(5′-GCGCGTCGTGAAGCGTTC-3′), universal-realtime-Reverse (5′-GTGCAGGGTCCGAGGT-3′); relative expression levels were normalized to those of the U6 snRNA.

### Dual-Luciferase Reporter Assay

Target gene were predicted online with TargetScan (http://www.targetscan.org/) and PicTar (www.pictar.mdc-berlin.de), and the 3′ UTRs of the predicted genes were cloned from mouse total cDNA, fused downstream of the coding sequence of the firefly luciferase, and ligated into the multiple cloning site (MCS) of the pcDNA3.1 plasmid. The miR-214 overexpression clone was ligated into the MCS of the pcDNA3.1 plasmid. Cells were seeded in 24-well plates 12 hours before transfection. Plasmids used were as follows: the Renilla luciferase expression vector (pRL-TK) (50 ng/well), pcDNA3.1-luciferase (200 ng/well), and the miRNA overexpression plasmid (600 ng/well). At 24 h post-transfection, cells were analysed using a Dual Luciferase Reporter Assay System (Promega). Renilla luciferase activity was used as a transfection control.

### Western blot analysis

Total protein was extracted from cultured cells with a protein lysis buffer (50 mmol/L Tris, pH 7.5, 150 mmol/L NaCl, 2 mmol/L EDTA, and 1% Triton X-100) supplemented with protease inhibitors (5 μg/mL phenylmethanesulfonyl fluoride, 2 μg/mL pepstatin, 2 μg/mL aprotinin, and 1 μg/mL leupeptin [Roche]). Lysates were cleared of insoluble material by centrifuging at 125,000 rpm at 4 °C. Supernatant were collected and boiled in SDS sample buffer, and SDS-PAGE gel electrophoresis was conducted according to the standard protocol, followed by western blotting. Antibodies used included the QKI antibody (ab126742, Abcam) and β-actin antibody (A5441, Sigma).

### *In utero* electroporation

For *in vivo* transfections, we generated miRNA and *QKI* overexpression constructs in the pCIG vector, provided by Wenmin Zhong (Yale University). The *QK*-shRNA sequence was synthesized as described previously^24^, and cloned into the pGPU6/GFP/Neo vector by Shanghai GenePharma Co, Ltd. All plasmids were extracted using the Endofree plasmid Maxi Kit (QIAGEN). *In utero* electroporation into timed pregnant CD-1 mice was performed as previously described^42^. Briefly, pregnant dams (E13.5) were anaesthetized by intraperitoneal injections with pentobarbital sodium, and the uterine horns were exposed, 3 μg/μl of plasmids spiked with Fast Green (Sigma) were injected into the lateral ventricle of the embryo brain. Electroporation was conducted with electric pulses of 30 V for 50 ms, which were repeated five times with 950-msecond intervals using the BTX-ECM830 electroporator (Harvard Apparatus).

### Immunohistochemistry

Immunohistochemical analyses of the cryosections and cells were conducted as previously described^43^. DAPI (ZLI-9557, ZSBio) was used for DNA staining to reveal the nuclei. The primary antibodies used for IHC were as follows: Pax6 (PRB-278P, Convance), Tbr2 (ab23345, Abcam), MAP2 (ab11267, Abcam), Nestin (ab22035, Abcam), and GFAP (ab7260, Abcam). Secondary antibodies included Alexa Fluor 488 (CA-11008, Molecular Probes) and 594 (CA-11005, Molecular Probes).

### Statistical Analysis

Statistical analyses were performed using Student’s t-test, with P values < 0.05 considered as statistically significant. For each comparison, the numbers from at least 3 individually transfected neocortices were averaged. Error bars represent the standard deviations or standard error from the mean.

## Electronic supplementary material

MicroRNA-214 modulates neural progenitor cell differentiation by targeting Quaking during cerebral cortex development
