# Supplementary material for: MicroRNA-214 modulates neural progenitor cell differentiation by targeting Quaking during cerebral cortex development
Source: Sci Rep. 2017 Aug 14;7:8014. doi: 10.1038/s41598-017-08450-8 (PMC5556025; doi:10.1038/s41598-017-08450-8)
Supplement: Supplementary file 1 — MicroRNA-214 modulates neural progenitor cell differentiation by targeting Quaking during cerebral cortex development [file 41598_2017_8450_MOESM1_ESM.doc]

**MicroRNA-214 modulates neural progenitor cell differentiation by targeting *Quaking* during cerebral cortex development**

**Pengcheng Shu^1#^, Hongye Fu^1#^, Xiangyu Zhao^1^, Chao Wu^1^, Xiangbin Ruan^1^, Yi Zeng^1^, Wei Liu^2^, Ming Wang^1^, Lin Hou^1^, Pan Chen^1^, Bin Yin^1^, Jiangang Yuan^1^, Boqin Qiang^1^, Xiaozhong Peng^1∗^**

^1^The State Key Laboratory of Medical Molecular Biology, Neuroscience Center and Department of Molecular Biology and Biochemistry

^2^Department of Anatomy and Histology

Institute of Basic Medical Sciences, Chinese Academy of Medical Sciences and Peking Union Medical College, Beijing 100005, China

**Supplementary Information**

**Supplemental Figure 1**


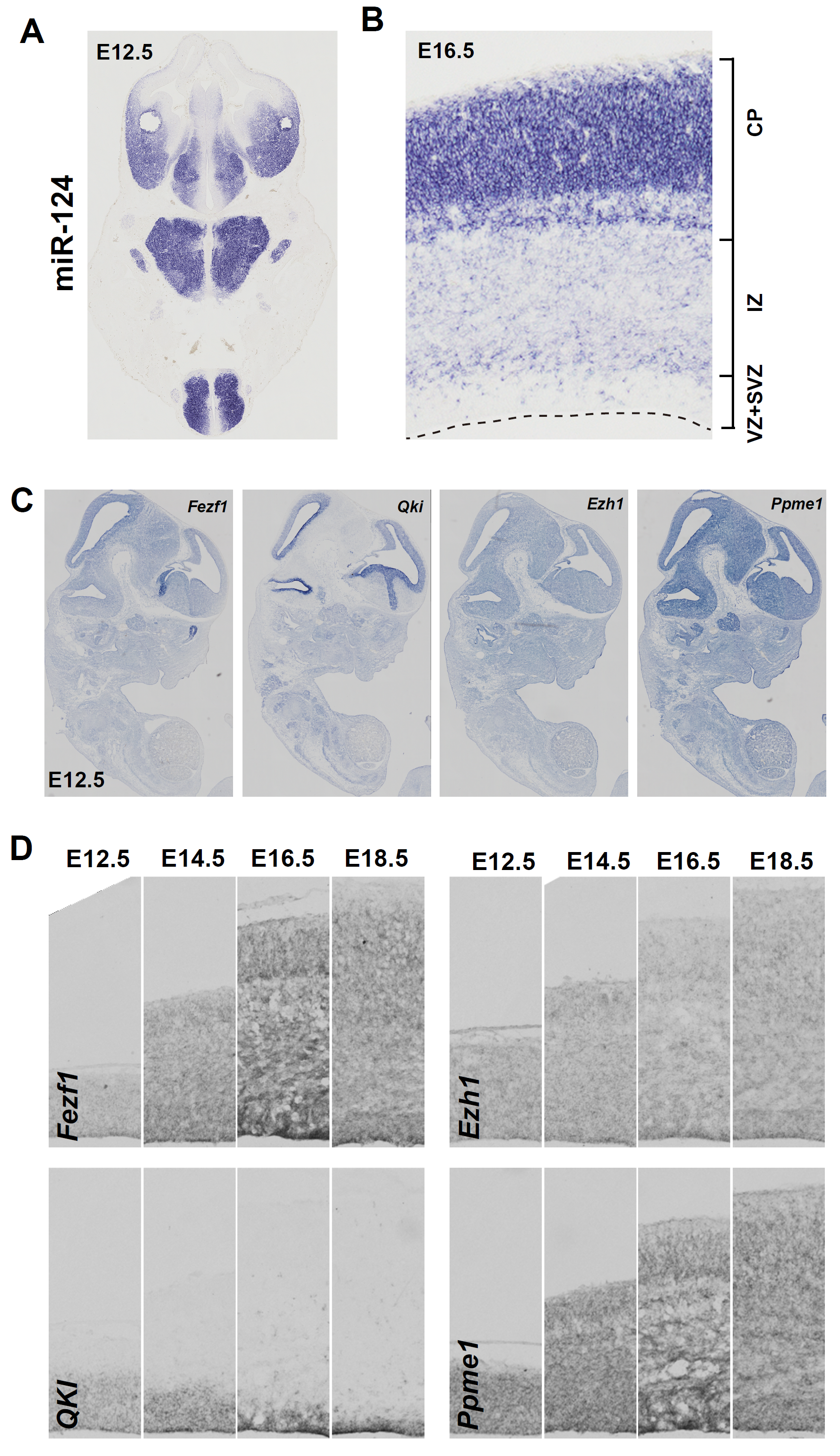


(A-B) The expression of miR-124 during cortical neurogenesis at E12.5 (A) and E16.5 neocortex (B). CP, cortical plate; IZ, intermediate zone; SVZ, subventricular zone; VZ, ventricular zone.

(C-D) The expression patterns of *Fezf1*, *Ezh1*, *Qki* and *Ppme1* during mouse embryonic development. *In situ* hybridization performed by specific probes. The dynamics of *Qki* mRNA expression in sagittal sections of mice whole body (C) at E12.5 and the coronal sections of cerebral cortex development at E12.5, E14.5, E16.5 and E18.5 (D) were shown.

**Supplemental Figure 2**


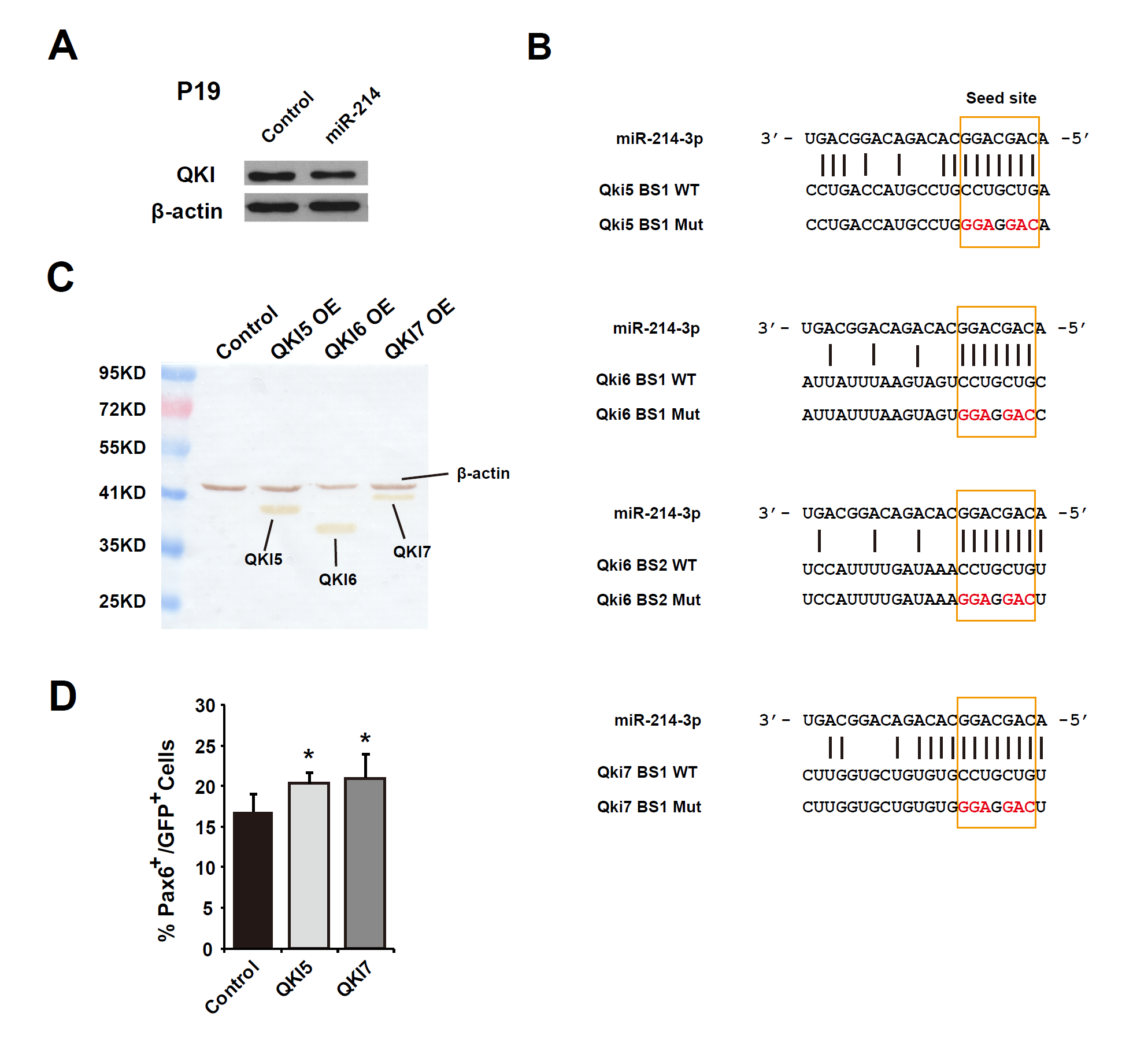


(A) Western blot analysis of QKI expression after overexpression of the miR-214 for 48 hours in P19 cells. β-actin was included as a loading control.

(B) The possible miR-214 binding sites (bs) predicted by TargetScan software (http://www.targetscan.org/). *Qki5* contains a miR-214 binding site, *Qki6* has two, and *Qki7* has three, the latter two of which are the same as Qki6. The 5’ seed sequences of miR-214 are marked by orange boxes. ‘WT’ stands for wild type and ‘Mut’ for mutant.

(C) Western blot examined the overexpression of the three *Qki* isoforms, is the naked eye result of the Figure 4A. The membrane firstly detected the expression of QKI, then β-actin after striping. The respective dark brown bands show the expression of QKI and β-actin after ECL substrate treated.

(D) Quantitative analysis of the E16.5 dorsal forebrains for the fraction of those co-expressing the radial glia marker Pax6 within the transfected neocortex shown in Figure 4E. Error bars show standard deviation, and the comparisons performed with Student’s t-test, the statistically significant P values shown as * (<0.05), ** (<0.01) or *** (<0.001).

**Supplementary Figure 3:** Uncropped versions of western blots.


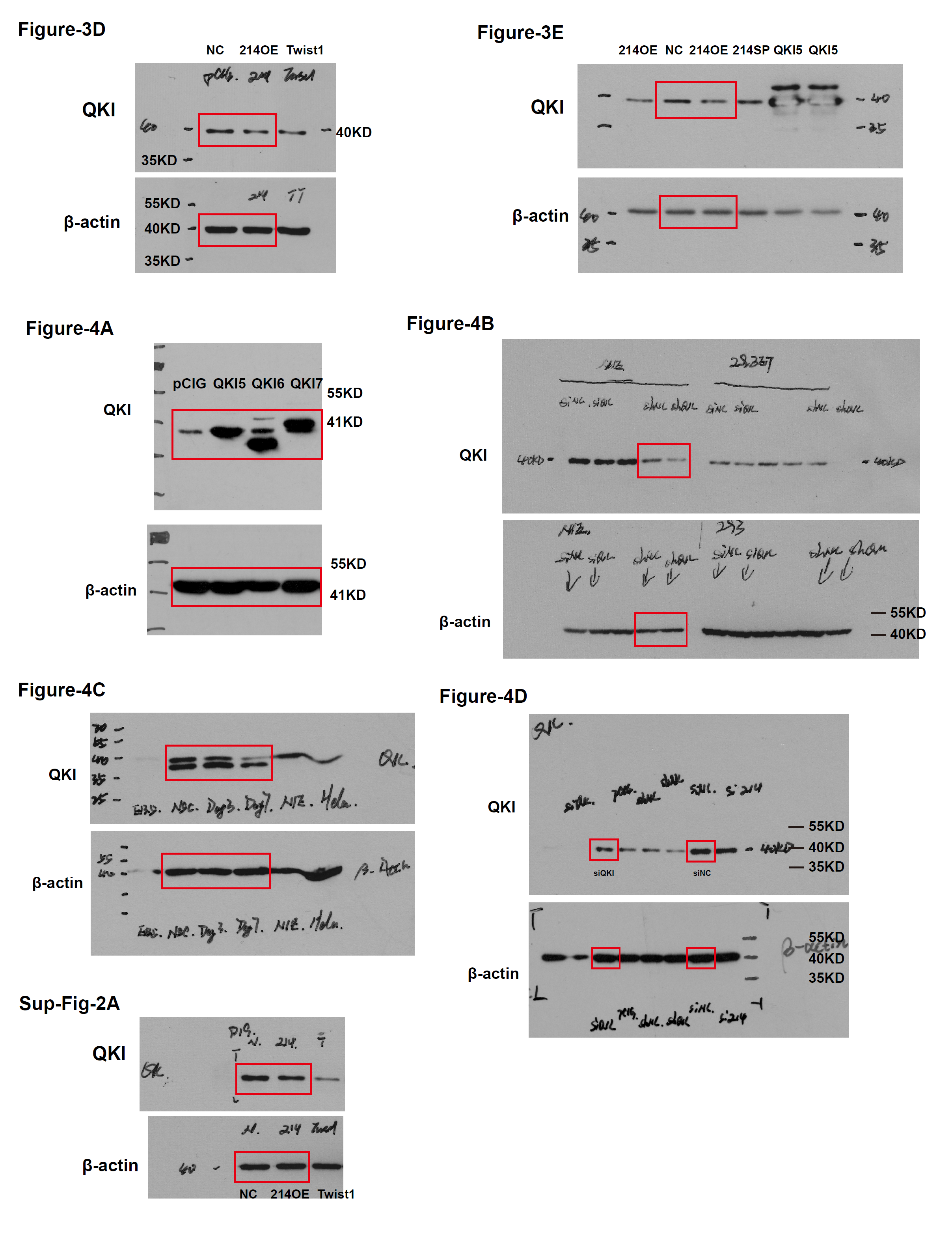


Full images of immunoblots shown in Figure 3D, 3E, 4A, 4B, 4C, 4D, Supplementary Figure 2A and Supplementary Figure 2B.
